# Strong wintering site fidelity contrasts with exploratory breeding site sampling in a socially monogamous shorebird

**DOI:** 10.1186/s40462-025-00580-3

**Published:** 2025-07-11

**Authors:** Eunbi Kwon, Mihai Valcu, Bart Kempenaers

**Affiliations:** Department of Ornithology, Max Planck Institute for Biological Intelligence, Eberhard-Gwinner-Strasse 8, 82319 Seewiesen, Germany

**Keywords:** Behavioral flexibility, Individual repeatability, *Limnodromus scolopaceus*, Long-billed dowitcher, Migration, Site fidelity, Breeding dispersal

## Abstract

**Background:**

The migration behavior of an organism is supposedly shaped by selection to best utilize favorable environmental conditions and unevenly distributed resources to maximize survival and reproductive success. Repeated migration tracks of individual birds allow us to estimate individual consistency in the spatio-temporal patterns of migration, and thereby better understand the potential constraints or drivers of migratory strategies.

**Methods:**

We caught 48 long-billed dowitchers (*Limnodromus scolopaceus*) on their nest in Alaska in 2019 and equipped them with a 2 g Solar Argos PTT-100 satellite transmitter. We obtained repeat migration data from 19 individuals (11 males, 8 females) for up to four years. First, we quantified the within-individual repeatability in migratory route and migratory timing during both southward and northward migration. Second, we defined the home ranges for breeding, staging and non-breeding sites for each individual, and assessed their spatio-temporal overlap across consecutive years.

**Results:**

Dowitchers were significantly more faithful to their wintering areas compared to any other stage of their annual cycle. Within their breeding range, individuals showed exploratory behavior and dispersed on average 159 $\:\pm\:$ 208 km (*N* = 42 bird-years) between breeding sites in consecutive years. The timing of migratory movements showed the highest individual repeatability when birds were at or near the wintering area.

**Conclusion:**

Our study demonstrates that the within-individual repeatability in spatio-temporal patterns of migration and site use in dowitchers varies across different stages of the annual cycle. The birds’ high fidelity to their wintering area contrasts sharply with a lack of fidelity to their breeding area. We suggest that the long-distance breeding dispersal – atypical for socially monogamous Scolopacids – is an adaptive response to unpredictable year-to-year variation in the physical and/or social environment during the breeding season.

**Supplementary Information:**

The online version contains supplementary material available at 10.1186/s40462-025-00580-3.

## Background

Twice a year, individuals of many bird species migrate over large distances. The spatio-temporal patterns of these movements depend on an individual’s ability to time its behavior through an endogenous circannual rhythm, its ability to navigate, and external cues that influence both timing and navigation [1, 2]. Advances in tracking technology have led to the accumulation of data on individual migration patterns across multiple years [3]. Such data on repeated migration enable measuring individual consistency in timing and space use before, during and after migration, and provide insights into potential drivers of within- and between-individual variation.

Low within-individual repeatability is often interpreted as behavioral flexibility that is favored by selection in response to an unpredictable environment [4]. Relevant environmental factors may include food availability, predation risk, risk of infection, and the availability of nest sites, roosts or mates, all of which could drive the evolution of flexible migration strategies including nomadism [5, 6]. However, it remains unclear whether high behavioral flexibility is part of a broader ‘behavioral syndrome’ that affects multiple aspects of migration or if individuals can show flexibility in some situations, while maintaining consistency in others.

In general, the migratory movements of an individual are often more consistent in timing than in spatial patterns [4, 7–10] but see [11, 12]. Furthermore, the timing of departure from the non-breeding area is generally more repeatable than the timing of arrival at the breeding site or the timing of post-breeding movements (reviewed in [13]). Although patterns of spatial repeatability vary between species (Table 1), several studies suggest that birds flexibly choose their migratory routes and stopover sites [7, 10, 14]. However, individuals typically return consistently to particular locations to breed [13, 15] or to spend the non-breeding season [16–18]. Nevertheless, breeding site fidelity can vary widely among related species and among populations of the same species (for example in the Ciconiiforms [19], Procellariiformes [20], and Charadriiformes [21]), as does fidelity to stopover and wintering sites [22, 23].

Site fidelity may be a general individual trait (e.g., linked to the ability to navigate, or to the age of the individual), or it may vary depending on the annual stage (e.g., due to variation in the costs or benefits of returning to the same area). So far, only a few studies have reported seasonal differences in within-individual repeatability of site use. For example, ferruginous hawks *Buteo regalis* showed high consistency in both their breeding and wintering sites, but were flexible in their fall stopovers and migratory routes [15]. In contrast, Eleonora’s falcons *Falco eleonorae* showed high individual repeatability in the use of particular stopover sites and barrier-crossings, especially males [24]. In many other studies, individuals either seem to be site faithful year-round (e.g. in Brown skua *Stercorarius antarcticus* [25], European cuckoo *Cuculus canorus* [26], Great reed warbler *Acrocephalus arundinaceus* [12], Lesser black-backed gull *Larus fuscus* [27], Marbled godwit *Limosa fedoa* [11], Osprey *Pandion haliaetus* [4], Red-throated loon *Gavia stellata* [28], Eurasian woodcock *Scolopax rusticola* [29]), or flexible in their choice of residency sites year-round (e.g. Marsh harrier *Circus aeruginosus* [4, 9], Scopoli’s shearwater *Calonectris diomedea* [30]).

Here, we assess the individual repeatability in spatio-temporal patterns of migration and in the use of residency areas in the long-billed dowitcher *Limnodromus scolopaceus*, a medium-sized, socially monogamous and relatively understudied shorebird that breeds along the Arctic coasts of north America and Russia and winters in the southern U.S. and Mexico [31]. Unlike most other socially monogamous scolopacid sandpipers, dowitchers showed an exceptionally low return rate to their previous breeding site [21, 32]. However, it remains unknown whether the lack of site fidelity is a general trait of dowitchers, and hence observed across annual stages or whether low site fidelity is a unique feature related to breeding. We tracked long-billed dowitchers throughout the year for up to four years per individual. The aim of this study is to estimate the within-individual repeatability in the migratory routes, the timing of migratory movements, and the site use at different stages of the annual life cycle.

**Table 1:** Examples of avian tracking studies that qualitatively or quantitatively reported within-individual repeatability in space use and in timing of annual migration. Tracking method: geo = geolocator, ptt = satellite transmitter, GPS = GPS transmitter. The measure of repeatability (*R*) is shown as a range when a study reported multiple values for subsets of data or for different variables. For studies that did not report a quantitative repeatability measure, we used three categories to describe whether individual birds were consistent or flexible in their spatio-temporal patterns of migration (consistent, flexible, mixed). “Mixed” refers to variation either within or among individuals. Species are sorted alphabetically. We identified the studies by searches in Google scholar with the keywords ‘bird migration’ and ‘repeatability’, and using backward citation searches

Species		Stage		Method	Repeatability	Source
**Spatial repeatability**						
Black-legged kittiwake *Rissa tridactyla*		non-breeding		Geo	consistent	[33]
Brown skua *Stercorarius antarcticus*		year-round		Geo	consistent	[25]
Common swift *Apus apus*		year-round		Geo	0–0.87	[34]
Common tern *Sterna hirundo*		wintering		Geo	0.97–0.99	[18]
Cory's shearwater *Calonectris borealis*		migration route		Geo	0.02–0.43	[7]
		stopover		Geo	Flexible	
		wintering & stopover		Geo	Mixed	[8]
Egyptian vulture *Neophron percnopterus*		migration route		GPS	0–0.29	[10]
Eleonora's falcon *Falco eleonorae*		year-round		GPS	0–1.00	[24]
Eurasian woodcock *Scolopax rusticola*		breeding		PTT	consistent	[29]
		wintering		PTT	consistent	
European roller Coracias garrulus		wintering		GPS	0.75–0.91	[35]
Ferruginous hawk *Buteo regalis*		breeding & wintering		PTT	0.65–0.73	[15]
		fall stopover & route		PTT	0.37	
Golden eagle *Aquila chrysaetos*		wintering		PTT/GPS	0.88–0.94	[36]
Great reed warbler *Acrocephalus arundinaceus*		year-round		Geo	0.59–0.99	[12]
Lesser black-backed gull *Larus fuscus*		year-round		GPS	Consistent	[27]
Long-tailed skua *Stercorarius longicaudus*		non-breeding		Geo	Mixed	[37]
Marbled godwit *Limosa fedoa*		year-round		PTT	Consistent	[11]
Marsh harrier *Circus aeruginosus*		migration route		PTT	0–0.46	[9]
		year-round		PTT	0–0.41	[4]
Northern gannet *Morus bassanus*		wintering		Geo	0.91–0.92	[17]
Oriental honey buzzard *Pernis ptilorhynchus*		spring stopover		PTT/GPS	1.00	[38]
		migration route		PTT/GPS	0.10–0.90	
Osprey *Pandion haliaetus*		spring migration		PTT	0.04–0.99	[4]
		fall migration		PTT	0.54–0.88	
Red-throated loon *Gavia stellata*		breeding		PTT	1.00	[28]
		non-breeding		PTT	0.54–0.98	
Round island petrel *genus Pterodroma*		breeding		Geo	consistent	[39]
		migration route		Geo	consistent	
Scopoli's shearwater *Calonectris diomedea*		year-round		Geo	0.14–0.32	[30]
Wood thrush *Hylocichla mustelina*		migration route		Geo	0.12	[14]
**Temporal repeatability**						
Black-legged kittiwake *Rissa tridactyla*		year-round		Geo	0.20–0.80	[33]
Black-tailed godwit *Limosa limosa*		spring migration		Geo	0.30–0.90	[40]
		fall migration		Geo	0.10–0.60	
		spring departure		Geo/PTT/GPS	0.78	[41]
		year-round		Geo/PTT/GPS	0.33–0.52	
Brown skua *Stercorarius antarcticus*		year-round		Geo	0.48–0.97	[25]
Common swift *Apus apus*		year-round		Geo	0–0.77	[34]
Common tern *Sterna hirundo*		breeding arrival		Geo	0.71–0.74	[18]
		breeding departure		Geo	0.57–0.72	
		wintering arrival		Geo	0.53–0.63	
Cory's shearwater *Calonectris borealis*		year-round		Geo	0.51–0.71	[8]
Egyptian vulture *Neophron percnopterus*		year-round		GPS	0.43–0.70	[10]
Eleonora's falcon *Falco eleonorae*		year-round		GPS	0–1.00	[24]
Eurasian woodcock *Scolopax rusticola*		breeding arrival		PTT	0.89	[29]
		winter departure		PTT	0.13	
European roller *Coracias garrulus*		year-round		GPS	0–0.57	[35]
Ferruginous hawk *Buteo regalis*		fall departure		PTT	0.69	[15]
		other		PTT	0.17–0.53	
Golden eagle *Aquila chrysaetos*		spring migration		PTT/GPS	0.76–0.85	[36]
		fall migration		PTT/GPS	0.33–0.34	
Great reed warbler *Acrocephalus arundinaceus*		year-round		Geo	< 0.41	[12]
Eurasian hoopoe *Upupa epops*		spring migration		Geo	0.24–0.43	[42]
		fall migration		Geo	0.73–0.75	
Icelandic whimbrel		spring departure		Geo	0.76	[43]
*Numenius phaeopus islandicus*		egg laying		Geo	0.11	
		migration		Geo	0.23–0.48	
Lesser black-backed gull *Larus fuscus*		year-round		GPS	0.51–0.77	[27]
Marbled godwit *Limosa fedoa*		year-round		PTT	0–0.50	[11]
Marsh harrier *Circus aeruginosus*		spring migration		PTT	0.33–0.99	[9]
		fall migration		PTT	0.45–0.55	
		spring migration		PTT	0.32–0.81	[4]
		fall migration		PTT	0.35–0.60	
Oriental honey buzzard *Pernis ptilorhynchus*		year-round		PTT/GPS	0.35–0.80	[38]
Osprey *Pandion haliaetus*		year-round		PTT	0.04–0.38	[4]
Purple martin *Progne subis*		year-round		Geo	0–0.39	[44]
Red-backed shrike *Lanius collurio*		migration		Geo	0–0.85	[45]
Red-throated loon *Gavia stellata*		breeding		PTT	0.40–0.55	[28]
		non-breeding		PTT	0.60–0.87	
Round island petrel *genus Pterodroma*		breeding arrival		Geo	0.81	[39]
		breeding departure		Geo	0.78	
Sabine's gull *Xema sabini*		breeding & wintering		Geo/PTT	flexible	[46]
		stopover arrival		Geo/PTT	0.5	
Scopoli's shearwater *Calonectris diomedea*		year-round		Geo	0.09–0.36	[30]
Wood thrush *Hylocichla mustelina*		spring migration		Geo	0.49–0.71	[14]
		fall migration		Geo	0.05–0.62	

## Methods

### Field methods

We studied a population of long-billed dowitchers that bred in Utqiaġvik (formerly Barrow), Alaska (71° 18′ N, 156° 44′ W) in June–July of 2019 ^32^. We captured a total of 48 incubating individuals (22 females and 26 males) by dropping a mistnet on the nest, and equipped them with a 2 g Solar Argos PTT-100 satellite transmitter (Microwave Telemetry Inc.) using a leg-loop harness made of medical-grade silicone tubing (No.14197, Reichelt Chemietechnik, Germany). The combined weight of the transmitter and harness was 2.6 g, which was approximately 2% of the bird’s body mass. Before releasing the bird, we took a 5–10 µl blood sample from the brachial vein and stored it in Queen’s lysis buffer. The sample was later used to determine the bird’s sex in the laboratory through PCR amplification with primers P2/P8 ^47^. All birds were released within 30 min from the time caught, and they showed normal behavior after release. All field procedures were approved by the Alaska Department of Fish and Game (permit number 20–146) and the U.S. Fish and Wildlife Service (permit number MB210494-1).

### Data filtering

The estimated location error of the satellite transmitters typically ranges between < 100–1500m^48^. Of all Argos locations (*N* = 50,882), 34% had an error radius ≤ 1500 m (classes 1, 2 and 3), 19% had an error radius > 1500 m (class 0), and 46% had no associated error radius estimation (classes A and B). Prior to analyses, we calculated the ground speed between every two sequential locations for each bird using the R package ‘geodist’ [49]. We then filtered out erroneous locations with ground speeds exceeding 150 km/h. Additionally, we visually inspected the data and manually removed any clearly erroneous locations. Overall, 11% of the data points were removed.

### Defining seasons

Based on the location data and the species’ known breeding and wintering distribution ranges (obtained from Birdlife International [50]), we defined four annual stages for each individual: breeding, southward migration, wintering, and northward migration. Separating between these stages was straightforward because the breeding and wintering ranges of long-billed dowitcher cover a relatively small latitudinal range and are well separated from each other.

We defined the ‘breeding season’ in a given year as the period starting when an individual entered the species’ breeding range and ending when the bird moved out of the breeding range. Similarly, we defined the ‘wintering season’ as the period starting when an individual entered the species’ wintering range and ending when the bird moved out of this range in the following spring. To identify whether individuals were within the breeding and wintering range, respectively, we used the R package ‘sf’ [51].

We defined the southward and northward migration period of each bird as the period between the departure from the breeding range and the arrival into the wintering range, and vice versa. Upon visual inspection of all location data, we identified one individual that spent the entire non-breeding season outside the species’ known wintering range. For this individual, we defined the start of the wintering season as the first date of residency apparent from clustered location fixes (see ***Defining residency areas and home ranges***) during the typical wintering period. Additionally, three individuals entered the known wintering range, but spent part of the non-breeding season outside of this range. For these individuals, we defined the end of the wintering season as the date on which the bird initiated a migratory flight north.

### Defining residency areas and home ranges

We identified residency areas during each stage, i.e. (1) staging or stopover sites (during southward and northward migration), (2) wintering sites, and (3) potential breeding sites, by using a density-based spatial clustering of applications with noise (DBSCAN) algorithm implemented in the R package clusterTrack [52–54]. This method requires three input parameters: the minimum number of points needed to form a cluster (MinPts), the radius ($\:\epsilon\:$, in meters) that defines the neighborhood of a point, and a temporal parameter (maxLag) that allows identifying temporally distinct clusters. We initially set the parameters to MinPts = 3, $\:\epsilon\:$ = 4000, maxLag = 10, and visually verified the output clusters against the raw location data. In some cases, individuals seemingly utilized a larger area, so we adjusted the parameters to MinPts = 5, $\:\epsilon\:$ = 8000, maxLag = 20. These two sets of parameters produced clusters that fit the data well, but in some cases, we further adjusted the parameters until the outcome matched the visual validation (see Table S1 in Supplementary Information).

For every identified cluster with a tenure > 1 day, we then (1) defined the 90% utilization distribution function (UD) (i.e., the smallest area where the probability to locate the individual equals 0.90) as the ‘home range’ (R package ‘adehabitatHR’ [55]), and (2) extracted a single location with the highest number of points per unit area (i.e., the location of the highest probability of occurrence) as the ‘center’ (R package ‘spatstat’ [56]). In total, we identified 841 residency areas, each of which contained on average 36 transmitted location points ($\:\pm\:$ 58, range = 3–504). Because the UD function requires a minimum of five data points, we duplicated data points for clusters that had less than five data points (N = 114 cases) before estimating the UD function.

### Migratory routes

To determine the migratory tracks, we first replaced all locations that belong to a particular cluster (i.e., a stopover site) by the center of that cluster for a given period. We then fitted a Move Persistence (‘mp’) model [57] to the dataset of each individual using the R package ‘AniMotum’ [58]. The model was used to create movement tracks based on predicted maximum likelihood locations every 15 minutes. We used a 15-minute interval because the predicted tracks more closely matched the observed locations at this interval compared to larger intervals such as 1 hour. We only used interpolated locations that were based on at least two observed locations within a 24 h period (for details, see [32]). We then defined each southward migration as starting on the last date an individual spent at the residency site with the longest tenure within the breeding range and ending when it arrived at the residency site with the longest tenure within the wintering range. Similarly, each northward migration was defined as starting on the last date an individual spent at the residency area with the longest tenure in the wintering range and ending when it arrived at the residency area with the longest tenure in the breeding range. Our technical definition of migration assumes the longest residency site within the breeding and wintering ranges to be the breeding site and the wintering site. We acknowledge that this definition could potentially include within-season dispersal movements as part of the annual migration—for example, if a dowitcher relocates to a new breeding site after a failed breeding attempt, or uses multiple short-term wintering sites. However, this inclusive definition aligns with our research objective of assessing individual repeatability in migratory movements, including the use of seasonally important sites.

### Temporal repeatability during migration

To estimate the within-individual repeatability in timing of advancing northward and southward migration, we first defined a set of 65 latitudinal bands of 1° (~ 111 km) width between 8°N and 73°N. These boundaries correspond to the southernmost (8.17°N) and northernmost latitude (72.67°N) reached by a dowitcher in our dataset. For each migratory track, we then identified the date of entry in each band (based on the interpolated data, see methods, ***Migratory routes***). For each 1° latitudinal band with data from at least two individuals, we calculated the repeatability, *R*, in arrival date using a Gaussian mixed model with ‘individual’ as random intercept, using the R package ‘rptR’ [59]. We estimated 95% confidence intervals for *R* through parametric bootstrapping over 100,000 iterations, and assessed statistical significance using likelihood ratio tests. The *p*-values were adjusted for multiple comparisons using the false discovery rate (FDR) method [60].

### Spatial repeatability in migratory route

To quantify spatial variation in migratory tracks within and among individuals, we first computed an overall ‘mean route’ by averaging the migratory tracks of all birds across all years into a single route, following the methods described in [61] and [27]. The resulting mean route consists of 500 points that span the full course of migration. These points are placed such that the distance to the nearest-neighbor locations on any of the actual tracks is minimized. We then calculated the longitudinal distance between each of the 500 points of the mean route and the nearest point on an individual track and averaged those distances for each 1° latitudinal band. This distance measure was arbitrarily assigned a negative value for distances to the west and a positive value for distances to the east of the population mean route. For each 1° latitudinal band, we calculated the repeatability *R* of this distance measure, as described above (see ***Temporal repeatability during migration***).

### Between-year fidelity to residency areas

For each of the four annual stages (i.e., breeding, south-bound stopover, wintering, north-bound stopover), we examined whether individuals showed consistency in site use by defining two variables: (1) the proportion of overlap calculated as the (total overlapped area $\:\times\:$ 2)/the sum of all the home ranges in a given stage in years x and x + 1, and (2) the minimum distance between the centers of any of the home ranges in a given stage in years x and x + 1. As individuals may have multiple residency sites in a given year, we considered the proportion of overlap for all areas combined for variable 1 and the two closest sites for variable 2.

We then tested whether individual consistency in site use differed among the four annual stages, using two generalized linear mixed effect models assuming a zero-inflated beta distribution for variable 1 (*model 1*) and a Gaussian distribution for variable 2 (*model 2*). Both models included the annual stage as the single explanatory variable and bird ID as a random effect (intercept). Models were fitted using the R package ‘glmmTMB’ [62].

### Temporal repeatability in arrival and departure dates at residency sites

We calculated the individual repeatability, *R*, for the arrival date at and the departure date from the breeding and wintering area, as well as for the start and the end of south-bound stopover. We excluded north-bound stopover from this analysis, because it typically occurs over a shorter period (median 5 days, range: 0.5–26 days, *N* = 60) and because only 7 individuals made a stop-over during north-bound migration in more than one year. Dowitchers show large between-individual variation in the duration of south-bound stopover [32]. Therefore, we also tested the within-individual repeatability in the duration of south-bound stopover between years.

All statistical analyses were conducted in R version 4.4.1 [63].

## Results

**Fig. 1:**
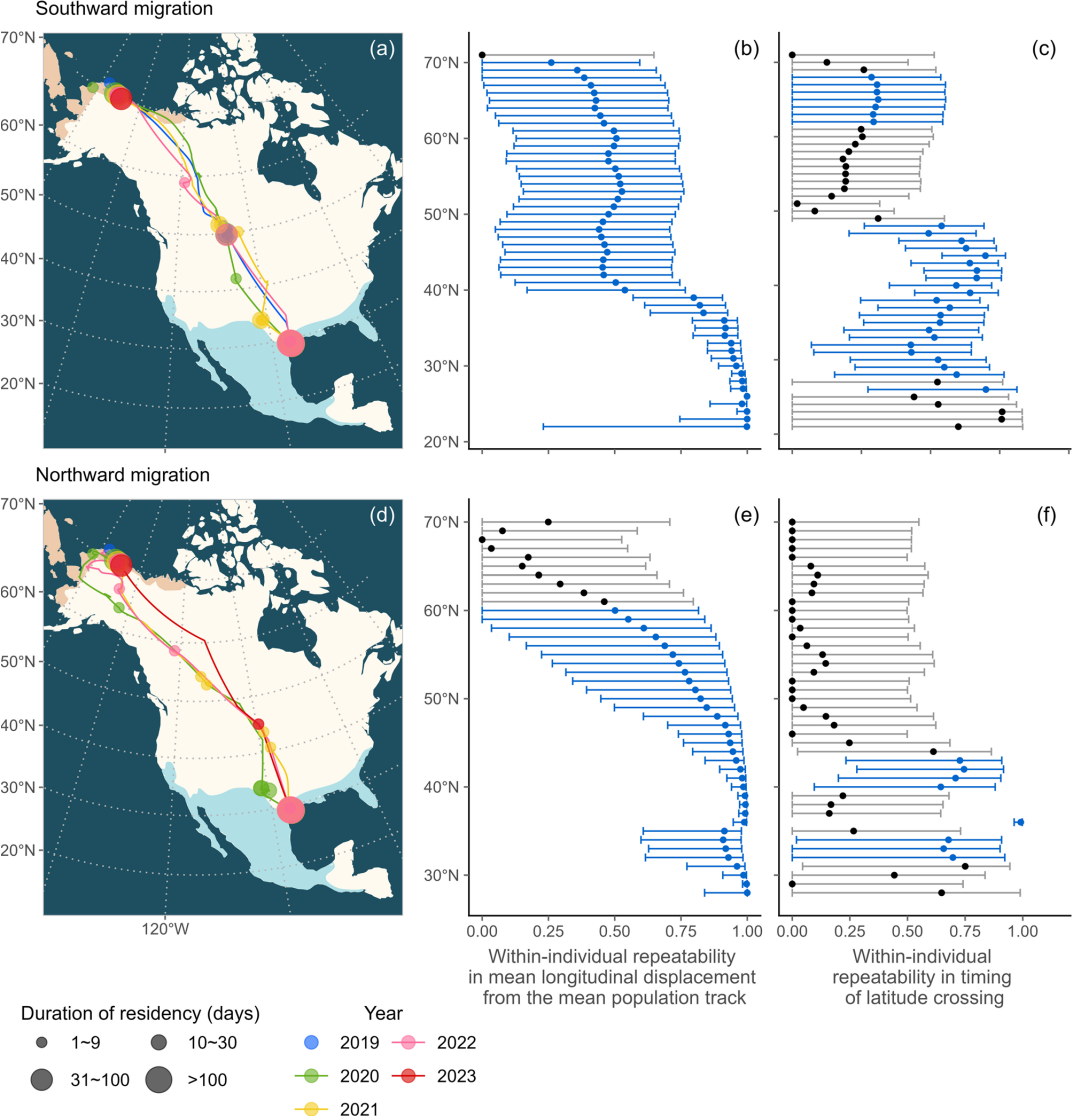
Migration tracks for an example long-billed dowitcher (male ID 66653; **a**, **d**) and within-individual repeatability of annual migration for 47 long-billed dowitchers based on satellite tracking from 2019 to 2023 (**b**, **c**, **e**, **f**). Panels (**b**, **e**) show spatial repeatability, while panels (**c**, **f**) display temporal repeatability between years. Point estimates of *R* and their 95% confidence intervals are presented for 1° (~ 111 km) latitudinal bands between 71°N and 25°N (less than five dowitchers wintered south of 25$\:^\circ\:$N). Statistically significant repeatability values are in blue. The migration tracks of all individuals can be found at http://ornithology.bi.mpg.de/ESM/Kwon_et_al_2025/

Repeated migration tracks were available for the southward leg in the autumn (*N* = 14, 3, and 2 dowitchers for 2, 3, and 4 consecutive years respectively) and for the northward leg in the spring (*N* = 6, 3, and 1 dowitchers for 2, 3, and 4 consecutive years respectively; Fig. S1). The wintering areas of the tracked individuals covered the longitudinal span of the species’ wintering range and overlapped between males and females (Fig. S2). Sixteen out of 19 dowitchers (10 males and 6 females) migrated through the Central Americas Flyway (see Fig. 1a, d for an example), two individuals (1 male and 1 female) used the Pacific Flyway [64] for both south- and northward migration, and one female switched flyways for the southward migration over a period of four years (ID 66746; see the Supplementary Maps). The sixteen individuals that migrated along the Central Americas Flyway staged for an average of 66 $\:\pm\:$ 30 days (*N* = 38 bird-years) between 32 and 59$\:^\circ\:$ N before continuing south (see the Supplementary Maps).

### Spatial repeatability in migratory route

The within-individual spatial repeatability of the migratory route was low (*R* < 0.5) at the beginning of southward migration until about 40$\:^\circ\:$N, where it abruptly rose and remained consistently high (*R* > 0.9) from 36$\:^\circ\:$N down to the wintering area (Fig. 1b). During the northward migration, the spatial repeatability stayed high (*R* > 0.9) until reaching 47$\:^\circ\:$N after which it steadily decreased as the birds continued their flight into the breeding range (Fig. 1e).

### Temporal repeatability during migration

**Fig. 2:**
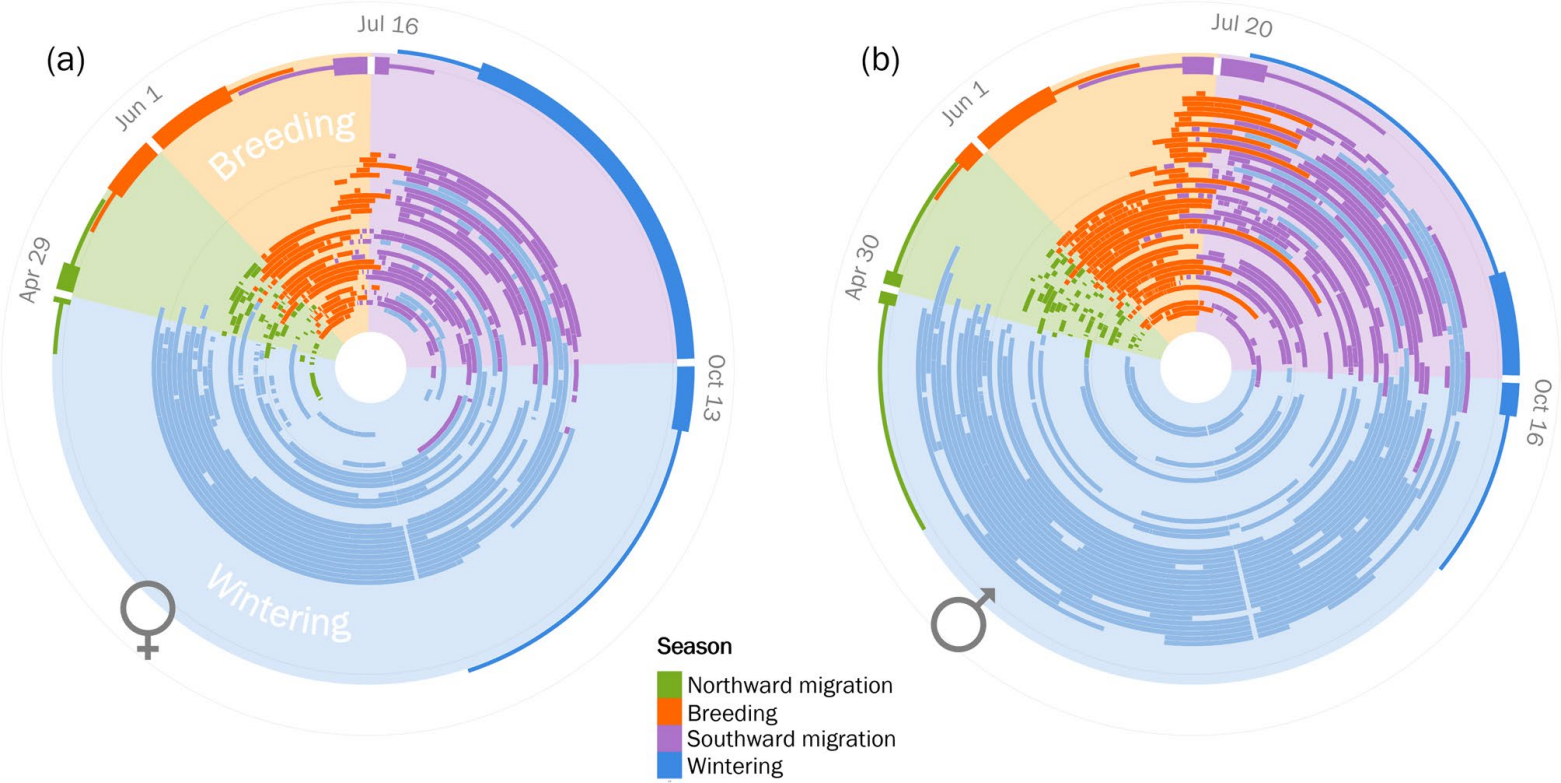
Variation in the annual timing of residency during different stages for 22 female (**a**) and 26 male (**b**) long-billed dowitchers derived from satellite tracking data collected from 2019–2023. Each part of a circle shows the timing of residency at a stopover (staging) site during northward (green) and southward migration (purple), and at a potential breeding (orange) and wintering site (blue) for a given individual in a given year (*N* = 32 female-years, *N* = 43 male-years). Summary boxplots (outer circle) indicate the minimum, 1st quantile, median, 3rd quantile, and maximum date for the start of each annual stage

The general timing of different stages of the annual life cycle was similar for male and female dowitchers. Dowitchers left the wintering area around 30 April (median, range: 15 March – 29 May, *N* = 30 individuals tracked up to 4 years, Fig. 2), first entered the species’ breeding range around 1 June (range: 12 May – 5 July), left the breeding range around 17 July (range: 20 June – 24 August), and arrived in the wintering range around 16 October (range: 21 July – 31 December). However, the timing varied more for females than for males (shown by the interquartile range in Fig. 2) except for the timing of departure from the breeding range (IQR = 10 days for females and 15 days for males; Fig. 2).

Individual between-year repeatability in timing was generally low (*R* < 0.37) at the onset of southward migration and further decreased until dowitchers reached around 49$\:^\circ\:$N (Fig. 1c). The between-year repeatability increased between 48$\:^\circ\:$N and 44$\:^\circ\:$N, and remained moderately high (*R* > 0.5) and significantly different from zero until reaching the wintering area (Fig. 1c). Individual repeatability in the timing of northward migration was in general lower than during southward migration and not significantly different from zero for most of the journey (Fig. 1f).

### Spatial repeatability in the use of residency areas

Individual dowitchers used on average 4.1 residency areas per year during the breeding seasons of 2020–2023 (range: 1−11, *N* = 29 birds, 172 areas), 3.5 areas for staging during southward migration (range: 1−9, *N* = 47 birds, 269 areas), 3.8 areas for wintering (range: 1−14, *N* = 40 birds, 241 areas), and 2.6 areas for staging during northward migration (range: 1−6, *N* = 29 birds, 108 areas; Table 2). The total area of the home ranges used by an individual in a given year was 203 km^2^ (median $\:\pm\:$ 262 SD) during the breeding season, 115 km^2^ ($\:\pm\:$ 156 SD) during south-bound staging, 187 km^2^ ($\:\pm\:$ 196 SD) during the wintering season, and 79 km^2^ ($\:\pm\:$ 96 SD) during north-bound staging (Table 2).

.

**Fig. 3:**
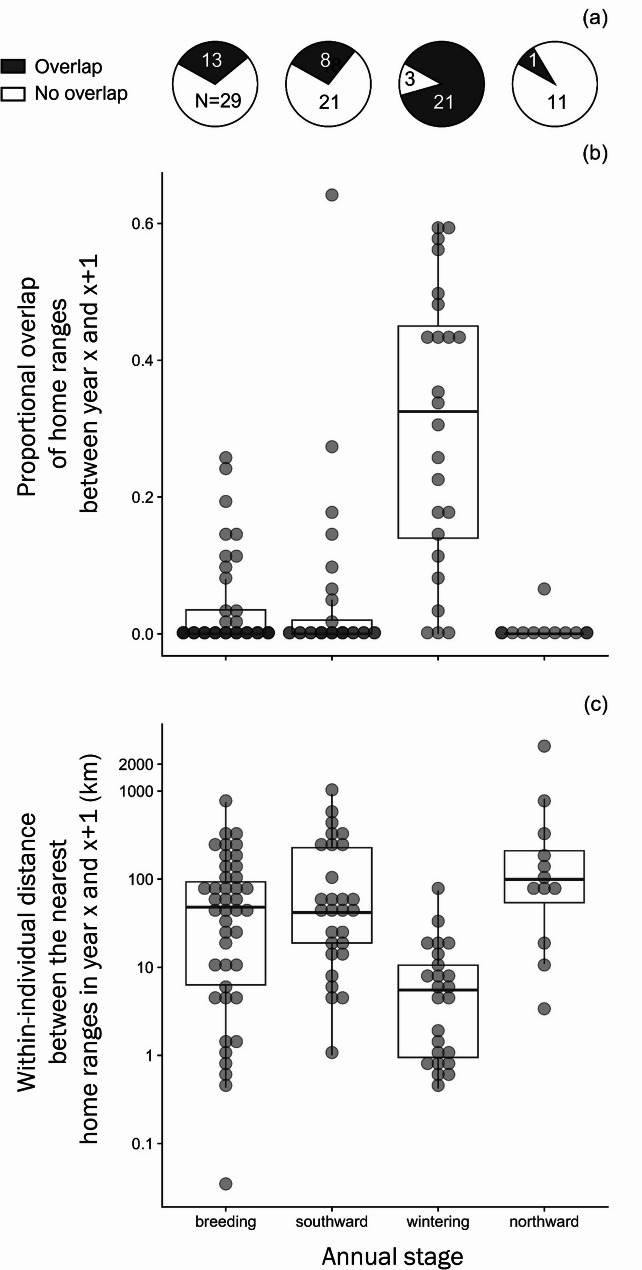
Spatial overlap of the home ranges (90% utilization distribution function) of individual long-billed dowitchers between two consecutive years based on satellite tracking data from 2019–2023 for different stages of the annual cycle (breeding, southward migration, wintering, and northward migration). (**a**) Pie charts show the number of cases (comparisons of the same individual between consecutive years, N = bird-years) where any home-range overlapped (yes/no). (**b**) The proportion of overlap between home ranges occupied by the same individual in two consecutive years within a given stage (1.0 indicates complete overlap). (**c**) The minimum distance between the center of the nearest home-ranges occupied by an individual dowitcher in two consecutive years within a given stage. Each dot indicates a bird-year data point (**b**, **c**)

**Fig. 4:**
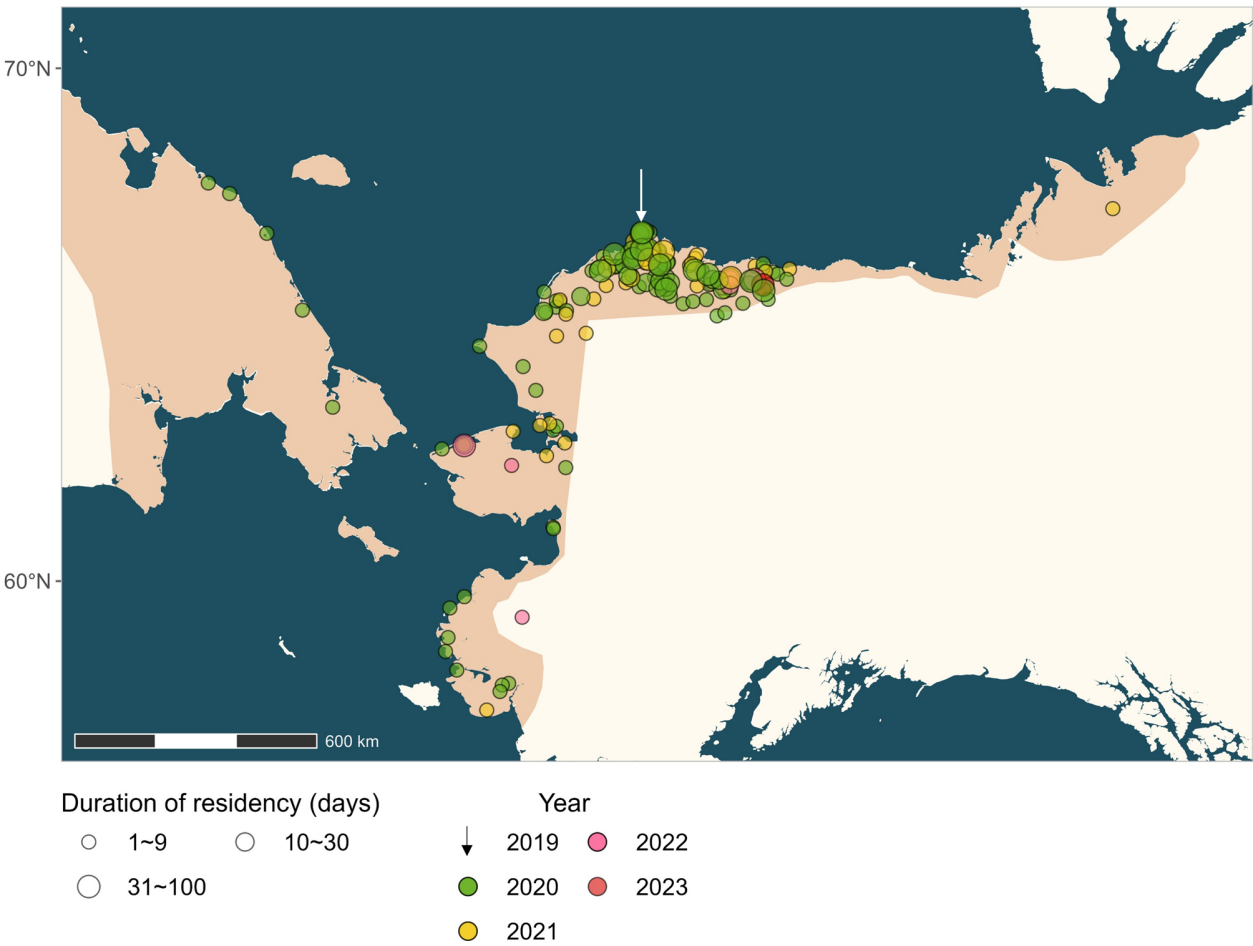
Residency areas of long-billed dowitchers during the breeding season derived from satellite tracking data collected from 2019–2023. White arrow: the deployment site near Utqiaġvik (Barrow) in 2019. The light orange area on the map depicts the known breeding range of the species

For 29 birds tracked for up to four years, the overlap in home ranges between subsequent years varied by stage. The home ranges overlapped in 31% of cases during breeding (*N* = 42 bird-year comparisons), in 27% of cases for south-bound staging (*N* = 29 bird-years), in 87% of cases during wintering (*N* = 24 bird-years), and in 0.8% of cases for north-bound staging (*N* = 12 bird-years Fig. 3a). The probability that the home range overlapped was significantly higher during wintering than during any other stage (zero-inflation part of model 1; Table S2). In addition, the non-zero proportional overlap was also significantly higher for wintering residency sites than for breeding and south-bound stopover sites (Fig. 3b, Table S2). The home ranges occupied by an individual in subsequent winters were also significantly closer together (average minimum distance between centroids = 10 $\:\pm\:$ 15 km SD, range: 0.4–73 km) than those occupied during any other annual stage (Fig. 3c, Table S3). When considering the longest breeding residency as the most likely nesting location for each bird and year, the mean breeding dispersal distance was 159 $\:\pm\:$ 208 km (range 0.03–1,187 km) between two consecutive years (Fig. 4).

**Table 2:** Summary of residency area metrics for long-billed dowitchers during different annual stages. Home range was defined as the 90% utilization distribution function (see Methods). Mean and median values are shown with standard deviation ($\:\pm\:$) and range (parentheses). Breeding residency data from the initial capture year of 2019 are excluded, because dowitchers may have staged at other residency areas prior to being tagged

	Breeding	South-bound stopover	Wintering	North-bound stopover
Number of individuals	29	47	40	29
Number of residency areas	172	269	241	108
Mean number of residency areas per individual & year	4.1 $\:\pm\:$ 2.2 (1–11)	3.5 $\:\pm\:$ 2.0 (1–9)	3.8 $\:\pm\:$ 2.8 (1–14)	2.6 $\:\pm\:$ 1.1 (1–6)
Mean number of transmitted location points per residency area	69.2 $\:\pm\:$ 89.8 (3–504)	21.8 $\:\pm\:$ 29.3 (3–261)	30.4 $\:\pm\:$ 40.7 (3–244)	12.9 $\:\pm\:$ 12.1 (3–78)
Median home range size (km^2^)	203 $\:\pm\:$ 262 (5–1,676)	115 $\:\pm\:$ 156 (0.9–1,449)	187 $\:\pm\:$ 196 (1–956)	79 $\:\pm\:$ 96 (1–806)

### Temporal repeatability in arrival or departure from residency areas

**Fig. 5:**
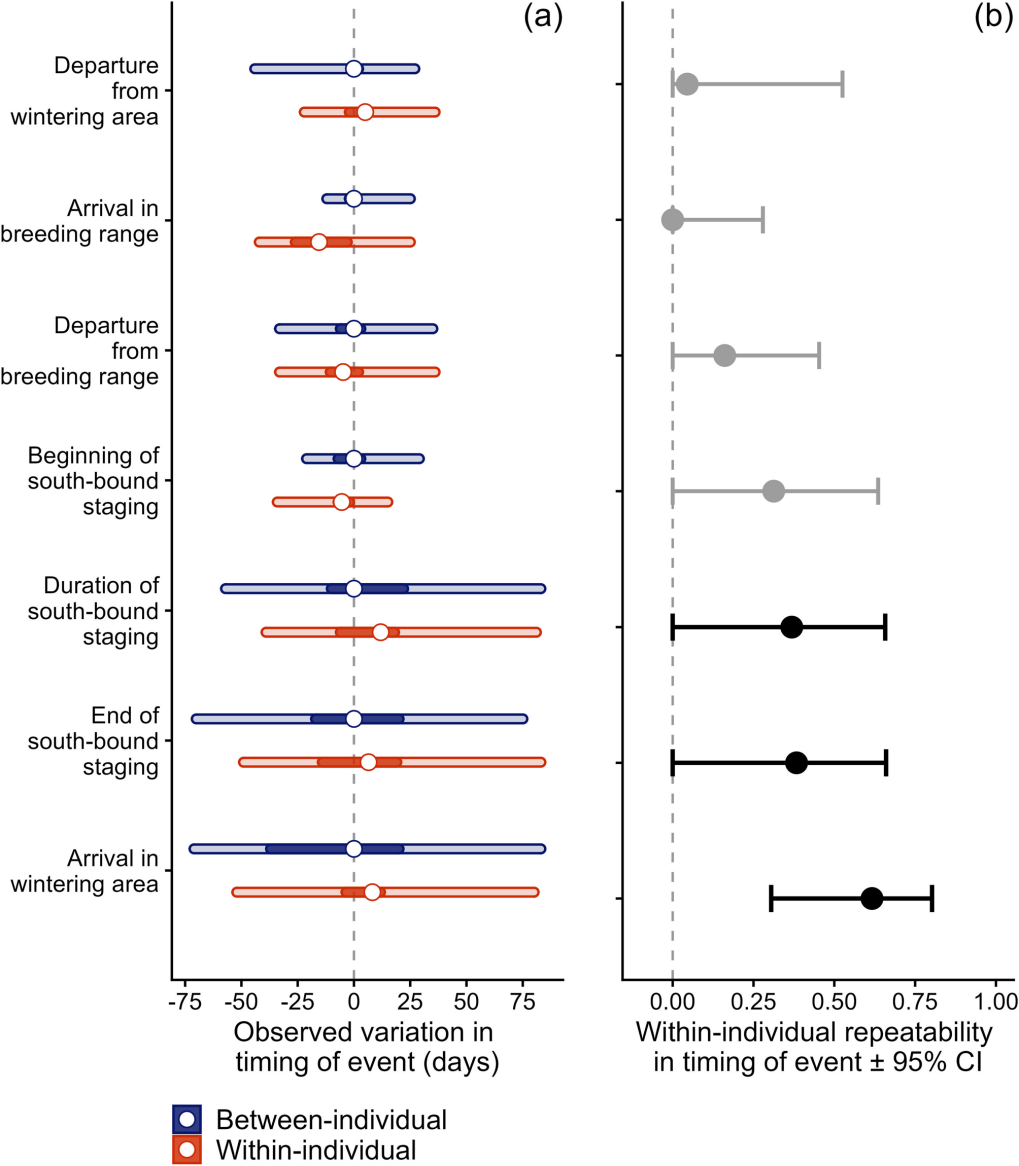
Observed within- and between-individual variation (**a**) and individual repeatability (**b**) in the timing of annual events for 48 long-billed dowitchers (22 females, 26 males) that were satellite-tracked between 2019–2023. (**b**) Between-individual variation is shown in blue, within-individual variation between years in red. Shown are the range (light-colored bar), the 1st and 3rd quantile (darker-colored bar), and the mean (open dot). To compare the between-individual variation for different annual stages, we subtracted the mean date for each event, such that the mean = 0. (**b**) The within-individual repeatability (R) in the timing of annual events is shown with its 95% confidence interval. Estimates that are significantly different from zero are shown in black. Long-billed dowitchers showed two stop-over strategies, with some individuals staying more than three months at a stop-over site, whereas others staying less than two weeks, causing large between-individual variation that carries over into subsequent stages. Note that three individuals switched between stop-over strategies across 4 years of observation

The duration of south-bound staging varied between 4 and 148 days (*N* = 70 bird-years). As a result, different individuals arrived at the wintering range up to 158 days apart (Fig. 5a). Among 20 dowitchers with available data, 3 switched between short (< 14 days) and long (> 70 days) south-bound staging in subsequent years. The remaining 17 individuals showed more consistent behavior: in subsequent years, they finished south-bound staging and arrived at the wintering area within on average 16 days (range: 1−40) and 12 (0−47) days, respectively. However, the within-individual repeatability was only significant for the timing of arrival in the wintering area (*R* = 0.62, 95% CI = 0.30–0.80; Fig. 5b). In contrast, within-individual repeatability for the timing of arrival in the breeding range between years was low, with overall lower variation among and within individuals (Fig. 5b).

## Discussion

Repeated migration tracks showed that individual long-billed dowitchers were relatively flexible in the timing of their migratory movements. Individuals showed strong fidelity to their wintering areas, but they were less consistent in their use of staging areas during migration, and they typically selected new breeding areas across years. Previous research showed a high breeding dispersal propensity in dowitchers, an unusual trait for a socially monogamous sandpiper [32]. Our study demonstrates that the degree of site fidelity varies across different stages of the dowitchers’ annual cycle, with high fidelity to the wintering area sharply contrasting with low fidelity to the breeding area.

Breeding site fidelity in long-billed dowitchers is extremely low compared to that of other socially monogamous shorebirds with biparental care [21]. The low breeding site fidelity of dowitchers may be the result of (1) weaker selection for fidelity to a breeding location, (2) stronger selection for flexibility in the choice of a breeding site, and (3) a behavioral syndrome in which individuals generally lack the urge or the navigational capability to return to the same site, possibly as a result of selection acting outside the breeding season. Long-billed dowitchers nest in moist-to-wet graminoid tundra habitat [65] and share many ecological traits with other sympatrically breeding, socially monogamous scolopacid shorebirds (e.g., a typical 4-egg clutch, bi-parental incubation with relatively fixed incubation bouts) [31]. However, unlike most other socially monogamous shorebirds, dowitchers show no mate fidelity [32]and they breed later in the season than other sympatric shorebirds [66]. These factors may have led to weaker selection on breeding site fidelity, because there is no advantage associated with meeting up with the breeding partner from the previous year and no advantage in terms of breeding earlier [66]. Alternatively, the lack of mate fidelity and later breeding may be the consequence of low site fidelity associated with a longer search for a suitable breeding area.

An earlier study [32] showed that long-billed dowitchers dispersed on average 172 km ($\:\pm\:\:$84 km SD, range = 45–321 km, *N* = 14 bird-years). Here, we increased the sample size and report a mean breeding dispersal distance of 159 km ($\:\pm\:$ 208 km, range = 0.03–1,187 km, *N* = 42 bird-years). This level of breeding dispersal may result from selection favoring flexibility in the choice of a breeding area. The stochastic nature of the timing of snow melt and between-year variation in local predation pressure in the high Arctic leads to unpredictability in the emergence of high-quality nesting habitat, which may favor such flexibility. However, the exceptionally low site fidelity of dowitchers suggests a species-specific environmental driver. A study on nest site selection showed that dowitchers prefer wet tundra habitat with some degree of topographic variation and taller vegetation within a 50-m radius of the nest [65]. The unpredictability of the occurrence of optimal nesting habitat across the Arctic, might favor exploratory movements within the breeding range.

Another important factor besides suitable nesting habitat is the availability of a mate. In our study area, a long-term breeding monitoring program showed that the annual nesting density of dowitchers fluctuated between 2.0 and 35.9 nests per km^2^ with a high coefficient of variation [66]. Such between-year variation in local densities may lead to unpredictability in mating opportunities at a given site, which may also drive exploratory movements and sampling multiple sites. Our tracking data show that dowitchers staged at more sites during the breeding season than in any other annual stage, and that the visited residency areas were quite broadly spread across the species’ known breeding range (see Fig. 4). This suggests that each year dowitchers sample multiple potential breeding sites and flexibly choose where to breed, which may also explain why they breed later in the season.

An alternative hypothesis is that dowitchers show less breeding site fidelity because they have reduced abilities to navigate to the same area, for example due to strong selection favoring nomadism during other parts of the annual cycle. In support of this hypothesis, the between-year site fidelity to migratory stop-over sites was also low (Fig. 3). However, two observations contradict the hypothesis. First, 31% of individuals (13/42) did revisit the same residency area during subsequent breeding seasons. Second, most individuals returned to the same wintering area (Fig. 3). These observations suggest that the lack of breeding site fidelity is an individual’s decision rather than a consequence of limitations in navigational capability and migratory homing behavior.

Long-billed dowitchers typically returned to the same general wintering area between years. The between-year individual repeatability of the migratory route increased as individuals approached their wintering range (Fig. 1), and their wintering home ranges showed considerable spatial overlap (Fig. 3). An extreme example was a dowitcher (ID 66746) that alternated between two flyways (the Central flyway in 2019/2021 and the Pacific flyway in 2020/2022), yet returned to the same site in California in all four winters (see Supplementary Map). This leads to the question why site fidelity is important during the non-breeding season, but not during other life stages. Many organisms tend to return to a home range or a territory, presumably because familiarity with a site leads to higher survival or fitness [67, 68]. Benefits of being site faithful include a “resident advantage” during territorial conflicts [69, 70], more efficient foraging [71, 72], a higher probability of escape from predators [73], and lower investment in aggressive behavior due to the “dear enemy” effect with known neighbors [74, 75]. Although these advantages of site familiarity may often apply during all annual stages, dowitchers only showed site fidelity during the non-breeding season (Fig. 3), i.e., during a period when they are presumably not territorial. Wintering site fidelity may still be beneficial if it improves foraging efficiency or predator avoidance, but it may also be related to the sparser, non-continuous distribution of suitable wintering habitat. Our transmitter location data suggest that most dowitchers stayed in areas with agricultural fields near a water body, such as an estuary or a river delta, or on small islands off the coast of the US and Mexico (see Supplementary Map). Recent tracking studies have shown extreme fidelity to a confined non-breeding staging area in other shorebirds (e.g., the mean home range size (90% or 95% UD) was 3.4 km^2^ [ for bar-tailed godwits *Limosa lapponica* [76], 5.5 km^2^ [ for Eurasian curlews *Numenius arquata* [77], and 12–17 km^2^ [ for dunlins *Calidris alpina* [78]). In our study, dowitchers used larger wintering home ranges of on average 187 $\:\pm\:$ 196 km^2^ [similar to what has been observed in the great knot *Calidris tenuirostris* (mean: 182 km^2^ [79]). Within the wintering range, water bodies may provide an easy landmark to revisit, but additional study is needed to elucidate the environmental variables that predict the wintering distribution of dowitchers and to assess the spatial limitations of optimal wintering habitat within the species’ wintering range.

Our tracking data clearly showed that individuals made extended stopovers during southward migration (Fig. 2). This observation suggests that dowitchers may molt during migration, a phenomenon that has been described for dowitchers staging in California on the Pacific flyway [80] but not for dowitchers using the Central flyway. Our results also suggest a sex difference in the timing of departure after breeding and subsequently in the timing of south-bound staging (Fig. 2). Females showed relatively little within-individual variation in the start of south-bound staging, but larger variation in the duration and the end of south-bound staging, whereas the trend was opposite for males. This pattern can be explained by our observation that females typically left the breeding area during late incubation or just after chick hatching, whereas males continued to care for the clutch or brood. Variation among males in the start of south-bound staging is then presumably due to differences in the timing of clutch or brood mortality.

Male and female dowitchers showed little fidelity to their south-bound staging areas. We hypothesize that three key factors play a role to explain the low site fidelity during this annual phase. (1) Given the low breeding site fidelity, individuals differ in the location from which they start their southward migration. (2) Individuals differ in their timing of onset of southward migration depending on the timing of breeding and breeding success, which may lead to differences in the availability of suitable staging areas. (3) Individuals vary in the timing of migration, but may have a fixed molt schedule such that they need to stop migrating when the time of molt approaches. The total duration of molt staging tended to be shorter if dowitchers left the breeding ground later in the season (Kempenaers, unpublished data). This observation fits to our finding that the temporal between-year repeatability during southward migration increased once dowitchers left the latitudes where molt staging presumably occurred. The sex-specific differences in the timing of southward migration and potentially in molt duration may therefore not propagate further into their annual cycle. In any case, only the timing of arrival at the wintering area was significantly repeatable between years (Fig. 5), although this may reflect the higher variance between individuals rather than the lower variance within an individual.

Most studies reporting on repeated migration tracks of individuals show that birds are either always flexible or always consistent in their choice of residency sites or migratory routes throughout their annual cycle (Table 1). Two studies showed stage-specific site fidelity and suggested that different underlying factors played a role. Ferruginous hawks showed post-breeding “seasonal nomadism”, presumably driven by seasonal limitations on fossorial prey and the hawks being free from nesting responsibilities and able to follow prey availability [15]. In Eleonora’s falcons, the spatial repeatability of migration was more related to geographic boundaries, seasonal variation in wind conditions and variation in an individual’s propensity for boundary crossing [24]. For relatively small-bodied shorebirds, data on tracking of movements across multiple years are scarce. Our literature search included two other shorebird species with year-round repeat tracking data, the marbled godwit and Eurasian woodcock (Table 1). In both studies, individuals showed site fidelity both during breeding and non-breeding stages of the year. For other shorebird species that breed sympatrically with dowitchers, such as the dunlin and the Pacific golden plover *Pluvialis fulva*, capture-resighting data suggest high fidelity to both wintering [81, 82] and breeding sites [21].

Variation in (breeding) site fidelity between species may also be related to mating strategies [19–21]. Numerous studies on birds have shown that social polygyny and polyandry are linked to sex-differences in the timing of migration (i.e., protandry and protogyny, respectively [83]). However, the role of the mating system and the intensity of sexual selection as potential drivers of migration programs remains underexplored, particularly in relation to spatial patterns. A recent comparative study on 186 bird species showed that migration distance correlated positively with mortality rates, male promiscuity, and divorce rate [84]. In pectoral sandpipers *Calidris melanotos* and ruffs *Calidris pugnax*, both socially polygynous/lekking species with intense male-male competition for matings and female-only care, most males traveled thousands of km within a single breeding season and visited multiple potential breeding sites [53, 85]. This seasonal nomadic behavior is presumably driven by mate availability [85]. Thus, although migration strategies have evolved under selection to minimize either the time or energy required to complete the migratory journey [86, 87], sexual selection may explain why individuals of some species move across large areas during a single season in search of mating opportunities, and therefore lack site fidelity [53, 85].

Our study shows that individual dowitchers visited on average 4 and up to 11 different potential breeding sites within a single breeding season. Thus, they sampled less than male pectoral sandpipers (on average 8 sites and up to 24) [86] and male ruffs (on average 11 sites and up to 23) [53, 85]. However, dowitchers form socially monogamous pairs with both pair members investing in incubation and they start breeding relatively late in the season. Thus, in contrast to polygamous species, it is highly unlikely that individuals of either sex can reproduce with multiple partners in a given season. Instead, we hypothesize that dowitchers benefit from exploring new areas by increasing their chances to find a suitable mate and an optimal nesting habitat, given generally low breeding densities and high between-year fluctuations in environmental conditions. To test this hypothesis, data on e.g. mate availability, breeding density and predation risk would be needed for all the sampled areas to compare their qualities as a breeding site. Unfortunately, such data are extremely difficult to obtain given the location of the breeding range. Under the paradigm of optimal migration theory, birds are expected to minimize the travel distance, or select a route that minimizes the energetic cost to reach the site of choice [86, 87]. Site fidelity may then be adaptive as the individuals accumulate experience to travel to this site [88]. We therefore predict that large-scale breeding site sampling has evolved under strong selective pressures. The challenge now remains to identify the selective forces that shape this behavior in dowitchers.

In conclusion, our study underscores the dynamic nature of migration with variation in spatio-temporal patterns within and between years. We show that site fidelity in long-billed dowitchers varies across different stages of their annual cycle. While individuals exhibit strong fidelity to wintering sites, they display greater flexibility in selecting breeding and staging locations. This flexibility may reflect a balance between the benefits of site familiarity and the need to adapt to fluctuating environmental conditions. To fully understand the evolutionary drivers of stage-specific site fidelity, future research should explore the interaction between natural and sexual selection, and how these forces influence migratory behavior throughout the annual cycle. Understanding how sexual selection may play a role in shaping species’ migration programs is important, not only to discover what may be an underappreciated driver of migration programs, but also to understand potential evolutionary constraints on migratory strategies and on the flexibility of such strategies. Such work should lead to insights into how individuals balance the trade-off between the benefits of familiarity to a particular area and the need for flexibility, for example in response to changing weather conditions and in response to perturbations in the landscape.

## Electronic supplementary material

Below is the link to the electronic supplementary material.

Supplementary Material 1

## Data Availability

The full dataset and the code are available at the Open Science Framework DOI: 10.17605/OSF.IO/RZNQC. The interactive maps of all migration tracks included in the study can be found at http://ornithology.bi.mpg.de/ESM/Kwon_et_al_2025/.
