# Supplementary material for: Strong wintering site fidelity contrasts with exploratory breeding site sampling in a socially monogamous shorebird
Source: Mov Ecol. 2025 Jul 11;13:49. doi: 10.1186/s40462-025-00580-3 (PMC12247350; doi:10.1186/s40462-025-00580-3)
Supplement: Supplementary file 1 — Supplementary Material 1 [file 40462_2025_580_MOESM1_ESM.docx]

**Supplementary material for ‘*Strong wintering site fidelity contrasts with exploratory breeding site sampling in a socially monogamous shorebird*’**

**Table S1.** Input parameters used for the function ‘tdbscan’ in the R package ‘clusterTrack’ to define spatiotemporal clusters (residency areas). MinPts: the minimum number of points needed to form a cluster, $\varepsilon$: the radius (in meters) that defines the neighborhood of a point, maxLag: a temporal parameter to split temporally distinct clusters.

| **Bird ID** | **Year** | **Season** | **minPts** | $\boldsymbol{\varepsilon}$ | **maxLag** |
| --- | --- | --- | --- | --- | --- |
| 66654 | 2020 | breeding | 5 | 10000 | 50 |
| 66687 | 2019 | South-bound staging | 5 | 10000 | 50 |
| 66688 | 2019 | wintering | 5 | 10000 | 50 |
| 66748 | 2020 | breeding | 5 | 7000 | 100 |
| 66748 | 2019 | South-bound staging | 5 | 7000 | 100 |
| 66832 | 2020 | wintering | 5 | 10000 | 100 |
| 66843 | 2020 | breeding | 5 | 4000 | 50 |
| 66844 | 2019 | wintering | 5 | 4000 | 50 |
| 66854 | 2020 | wintering | 5 | 7000 | 100 |
| 66857 | 2020 | wintering | 5 | 4000 | 70 |
| 66858 | 2020 | breeding | 5 | 5000 | 70 |
| 66861 | 2020 | breeding | 5 | 5000 | 70 |
| 66862 | 2020 | breeding | 5 | 6000 | 70 |
| 66862 | 2019 | wintering | 5 | 7000 | 100 |
| 66864 | 2020 | breeding | 5 | 7000 | 100 |

**Table S2.** Estimates for stage-specific proportional overlap of the core use area (50% utilization distribution function) of individual long-billed dowitchers between two consecutive years based on satellite tracking data from 2019–2023 for the breeding, south-bound & north-bound migration, and the wintering period. The wintering area was set as the reference (‘Intercept’). Because the proportional overlap values were bound between 0 and 1, we fitted a generalized linear model with a beta distribution (conditional model). “Stage” was included as the single explanatory variable and bird ID as a random effect. Because our data included many zeros, we additionally tested whether the probability of zeros differed among annual stages (zero-inflation model).

|  | Estimate | SE | z | *p* |
| --- | --- | --- | --- | --- |
| **Conditional model** |  |  |  |  |
| (Intercept) | -0.717 | 0.179 | -4.013 |  |
| Breeding area | -1.160 | 0.285 | -4.073 | >0.001 |
| South-bound stopover | -0.984 | 0.328 | -3.000 | 0.003 |
| North-bound stopover | -1.454 | 0.909 | -1.600 | 0.110 |
|  |  |  |  |  |
| **Zero-inflation model** |  |  |  |  |
| (Intercept) | -1.946 | 0.617 | -3.153 | 0.002 |
| Breeding area | 2.748 | 0.702 | 3.917 | >0.001 |
| South-bound stopover | 2.911 | 0.744 | 3.912 | >0.001 |
| North-bound stopover | 4.344 | 1.213 | 3.580 | >0.001 |
|  |  |  |  |  |
| **Random effect** | Variance | SD |  |  |
| ID | 0.150 | 0.387 |  |  |
|  |  |  |  |  |
| Number of observations = 107, groups = 29 | | | | |
|  |  |  |  |  |

**Table S3.** Estimates of the minimum distance between the centroids of the nearest core use areas used by individual long-billed dowitchers within a given annual stage in two consecutive years based on satellite tracking data from 2019–2023. The wintering area was set as the reference stage (‘Intercept’). We fitted a linear mixed effect model with “stage” as the single explanatory variable and with bird ID as a random effect. The dependent variable was log-transformed prior to model fitting.

| **Fixed effect** | Estimate | SE | z | *p* |
| --- | --- | --- | --- | --- |
| (Intercept) | 1.378 | 0.372 | 3.708 | >0.001 |
| Breeding area | 1.829 | 0.466 | 3.926 | >0.001 |
| South-bound stopover | 2.475 | 0.507 | 4.887 | >0.001 |
| North-bound stopover | 3.200 | 0.644 | 4.971 | >0.001 |
|  |  |  |  |  |
| **Random effect** | Variance | SD |  |  |
| Id | 4.407e-08 | 0.0002 |  |  |
| Residual | 3.315 | 1.821 |  |  |
|  |  |  |  |  |
| Number of observations = 106, groups = 29 | | | | |
|  |  |  |  |  |

**Figure S1.** The number of long-billed dowitchers whose satellite transmitters provided location data for different stages during the annual cycle from 2019–2023.

**
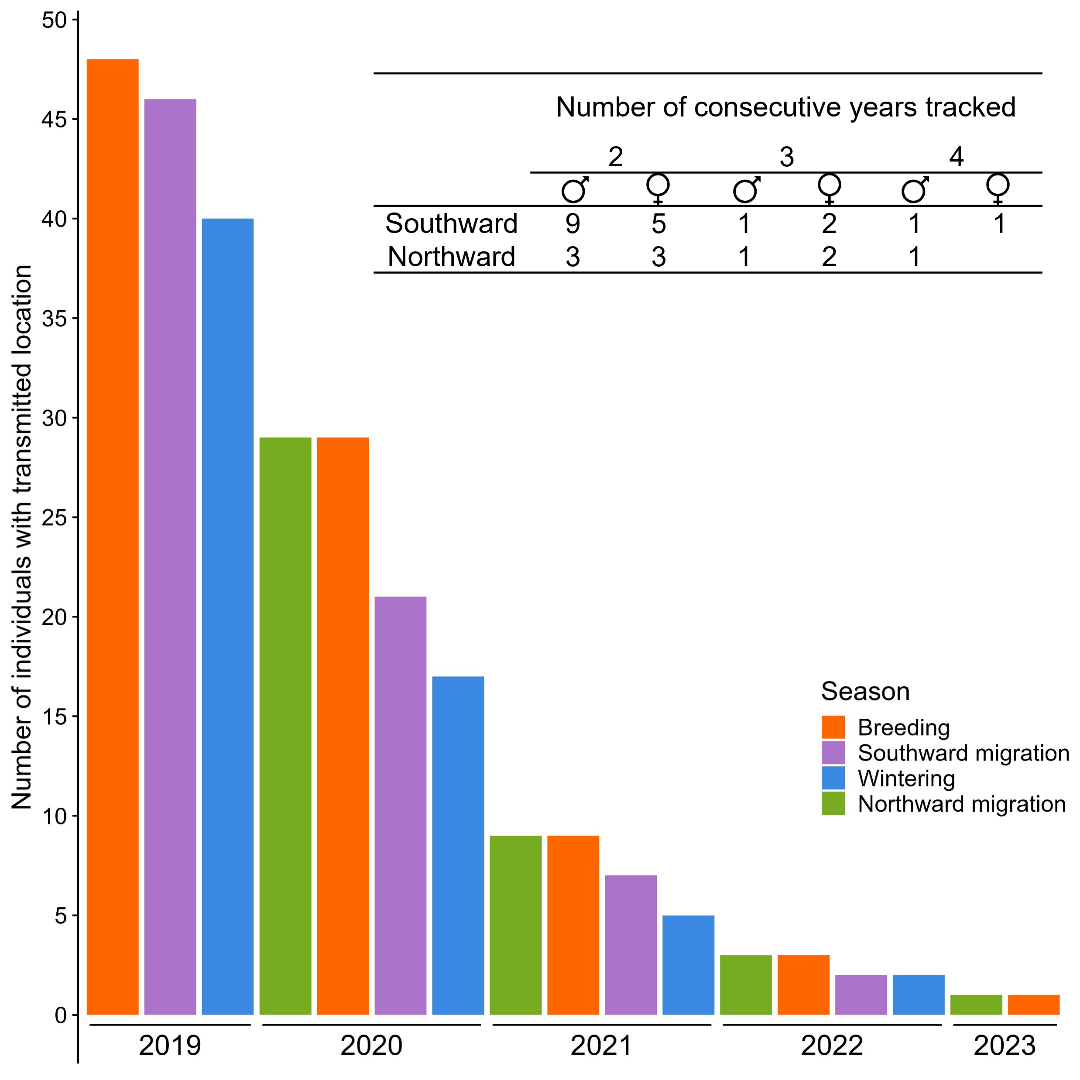
**

**Figure S2.** Interpolated southward (a) and northward (b) migration tracks of 47 long-billed dowitchers based on satellite tracking between 2019–2023 (gray) and the overall population mean route (black). Note that the mean route does not represent the most frequently used migration path, but only serves as a reference for calculating spatial repeatability (see *Methods: Spatial Repeatability in Migratory Route*).

**
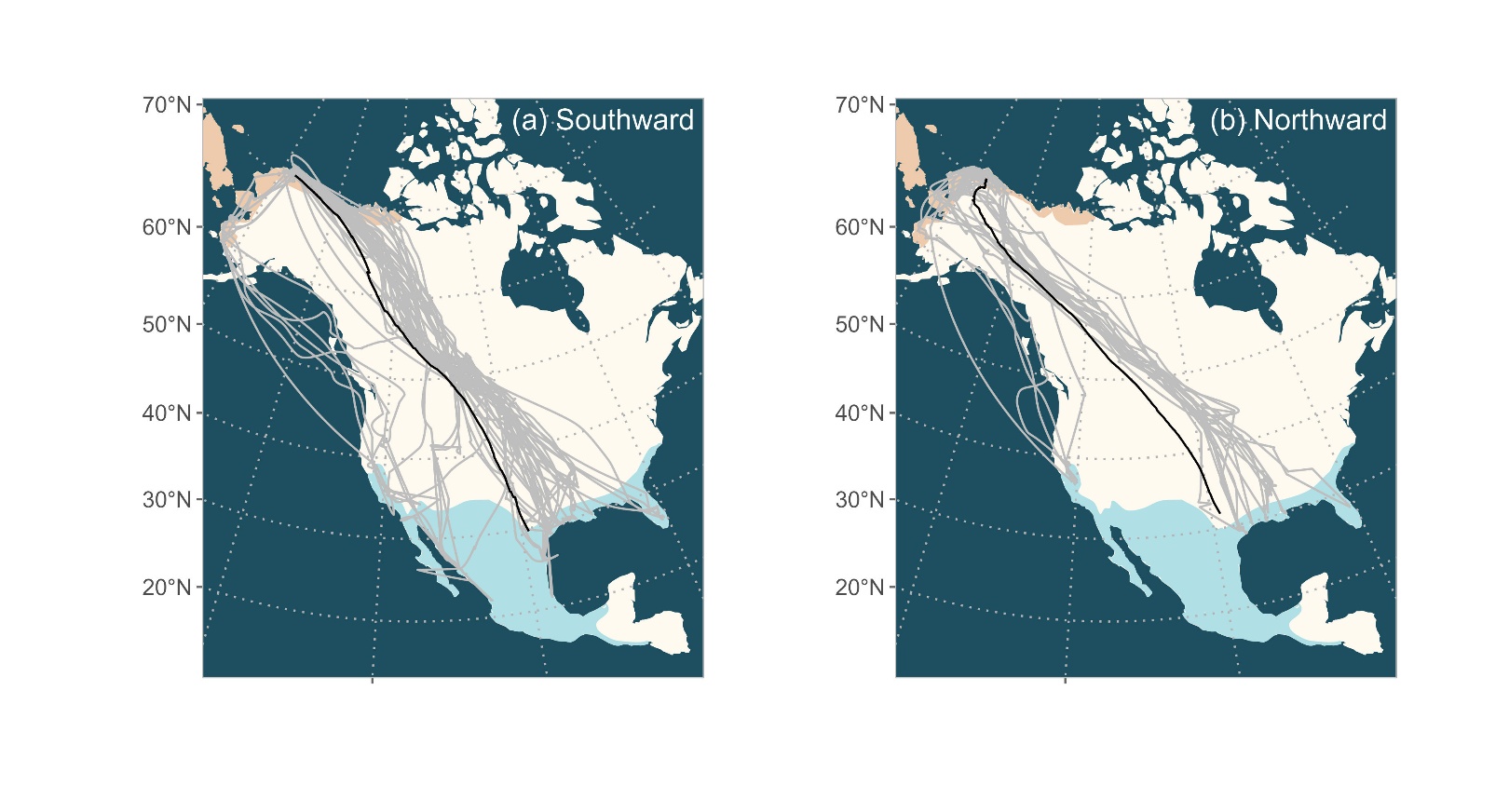
**

**Supplementary Map**: The interactive maps of migration tracks of all individuals can be found at http://ornithology.bi.mpg.de/ESM/Kwon_et_al_2025/
